# Co-expression of t(15;17) and t(8;21) in a Case of Acute Promyelocytic Leukemia: Review of the Literature

**DOI:** 10.4274/Tjh.2012.0180

**Published:** 2013-12-05

**Authors:** Burak UZ, Eylem Eliaçık, Ayse Işık, Salih Aksu, Yahya Büyükaşık, İbrahim C. Haznedaroğlu, Hakan Göker, Nilgün Sayınalp, Osman İ. Özcebe

**Affiliations:** 1 Hacettepe University Medical School, Department of Internal Medicine, Division of Hematology, Ankara, Turkey

**Keywords:** Additional chromosomal abnormalities, Acute promyelocytic leukemia

## Abstract

Additional chromosomal abnormalities in acute myelogenous leukemia have been identified as one of the most important prognostic factors. Favorable chromosomal changes such as t(8;21), inv(16), and t(15;17) are associated with higher rates of complete remission and event-free survival. Translocation (15;17) characterizes acute promyelocytic leukemia (APL) (French-American-British class M3) in almost all patients. Secondary chromosomal abnormalities are also present in approximately 23%-29% of patients with newly diagnosed APL. The prognostic implications of t(8;21) and other secondary cytogenetic aberrations in APL are reviewed here. We present a 47-year-old woman diagnosed with APL whose initial cytogenetic analysis included both t(8;21) and t(15;17). The initial induction chemotherapy included 3 days of idarubicin (12 mg/m2/day) and daily all-trans retinoic acid (ATRA; 45 mg/m2/day). At the sixth week of treatment, a control bone marrow biopsy was found to be normocellular, t(15;17) bcr3 and t(8;21) were negative, and t(15;17) bcr1 fusion transcripts were reduced from 5007 (1.78525699%) copies per 1 µg RNA to 40 (0.00062020%) with real-time quantitative polymerase chain reaction. Consolidation with 4 days of idarubicin (5 mg/m2/day), ATRA (45 mg/m2/day for 15 days), and cytarabine (1 g/m2/day for 4 days) was then started. However, the patient became pancytopenic and had neutropenic fever after consolidation treatment. Unfortunately, she died 3 months after the time of APL diagnosis, due to acute respiratory distress syndrome-like respiratory problems and multiorgan dysfunction requiring respiratory support and hemodialysis.

**Conflict of interest:**None declared.

## Abstract (tr)

Akut miyeloid lösemide ek kromozomal anomaliler en önemli prognostik faktörlerden biri olarak belirlenmiştir. Translokasyon t(8;21), inv(16), ve t(15;17) gibi olumlu kromozomal değişiklikler daha yüksek tam remisyon ve olaysız sağkalım ile ilişkilidir. Translokasyon (15;17) neredeyse tüm hastalarda akut promiyelositik lösemiyi (APL) tanımlamaktadır (Fransız-Amerikan-İngiliz [FAB] sınıf M3). Yeni tanı alan APL hastalarının yaklaşık %23-29’unda ikincil kromozomal anomaliler de bulunmaktadır. Bu olgu vesilesiyle APL’de t(8;21) ve diğer ikincil sitogenetik aberasyonların prognostik etkileri gözden geçirilmiştir. APL tanısı konan 47 yaşındaki kadın hastanın tanı anındaki sitogenetik analizinde t(8;21) ve t(15;17) birlikteliği saptandı. Başlangıç indüksiyon kemoterapisi 3 gün idarubisin (12 mg/m2/gün), ve günlük all-trans retinoik asit (ATRA) (45 mg/m2/gün) içermekteydi. Tedavinin 6. haftasında yapılan kontrol kemik iliği biyopsisi normoselülerdi. t(15;17) bcr3 ve t(8;21) negatifti, ve gerçek zamanlı kantitatif polimeraz zincir reaksiyonu (RQ-PCR) ile tespit edilen t(15;17) bcr1 füzyon transkriptleri 5007 (1,78525699 %) kopya/1 µg RNA’dan 40 (0,00062020 %)’a düşmüştü. Bunun üzerine 4 gün idarubisin (5 mg/m2/gün), 15 gün ATRA (45 mg/m2/gün), ve 4 gün sitarabin (1 g/m2/gün) içeren konsolidasyon tedavisi başlandı. Konsolidasyon tedavisi sonrasında hastada pansitopeni ve nötropenik ateş tablosu gelişti. APL tanısından 3 ay sonra, solunum desteği ve hemodiyaliz gerektiren çoklu organ fonksiyon bozukluğu ve ARDS-benzeri solunum problemleri nedeniyle hasta kaybedildi.

## INTRODUCTION

Acute promyelocytic leukemia (APL) accounts for approximately 10%-15% of acute myelogenous leukemia (AML) cases and is a very specific subtype with regard to morphologic, clinical, and prognostic features [W]. Although APL has the highest frequency of hemorrhagic morbidity and mortality among all subtypes of AML, most patients with t(15;17) will respond to a combination treatment with all trans-retinoic acid (ATRA), cytarabine (Ara-C), and anthracycline-based chemotherapy [1,2]. 

Balanced chromosome rearrangements are detected in approximately 25%-30% of adults with de novo AML [3,4,5] and have attracted a great deal of attention because of specific translocations and inversions associated with the prognosis of the patients harboring them. t(15;17) is known to be found in almost all APL patients [1]. Chromosomal abnormalities accompanying t(15;17) are reported in 23%-39% of APL cases [2,6,7,8,9,10]. Additional chromosome rearrangements including t(8;21) and t(15;17) are rarely seen in APL patients [11,12]. However, the clinical impact of these secondary anomalies has not been clearly elucidated yet. 

Herein, we present an APL patient who had both t(15;17) and t(8;21) at the time of diagnosis. Diagnosis of APL in this patient was based on karyotyping, immunophenotyping, and molecular studies of bone marrow cells. The treatment and clinical course of the patient is summarized. Informed consent was obtained. 

## CASE REPORT

In February 2012, a 47-year-old woman was admitted to the General Surgery Department of our institution with a 3-week history of fever, anal pain, and rectal discharge. Her initial blood count showed pancytopenia as follows: Hb, 55 g/L (normal range: 117-155), WBC: 1.6 x 10^9^/L (normal range: 4.1-11.2) with 0.2 x 10^9^/L absolute count (10.4%) of neutrophils, and platelets, 56 x 10^9^/L (normal range: 159-388). Peripheral blood smear revealed 1% neutrophils, 11% lymphocytes, 88% lymphomononuclear cells, poikilocytosis, anisocytosis, and schistocytes. Besides a mild increase in fasting blood glucose (6.56 mmol/L; normal range: 3.9-6.1), all biochemical parameters were within normal limits. Abdominal magnetic resonance imaging (MRI) revealed a lesion consistent with abscess formation (1.8 x 1.3 cm) adjacent to the right of the anal canal, which was hyperintense in T2A-weighted images and demonstrated peripheral involvement after the administration of intravenous contrast medium. A lateral internal sphincterotomy was performed in March 2012. After the operation she had continuous fever and leukocytosis (21.8 x 10^9^/L) with 19.6 x 10^9^/L absolute count (89.9%) of neutrophils. Her platelet count decreased to 11 x 10^9^/L. Despite replacement with platelet suspensions, she had retinal bleeding. Coagulation parameters revealed a increased prothrombin time (2.22 international normalized ratio; normal range: 0.86-1.20), D-dimer level (>20 mg/mL; normal range: 0-0.48), and thrombin time (27.2 s; normal range: 16.6-22.5). Fibrinogen level was found to be low (1.4 µmol/L; normal range: 6.4-11.9). Activated partial thromboplastin time was normal. The patient was referred to the hematology division. Her physical examination revealed fever (38.3 °C), generalized petechiae, and bilateral complete loss of pupillary light reflex. She did not have a chronic disease or history of drug usage. She had been pancytopenic since December 2011, and a bone marrow biopsy performed at another center was found to be normocellular (50%), with no finding of dysplasia. The bone marrow biopsy was repeated to reveal a marked increase in cellularity, with a grade I-II/III increase in reticulin network. Intermediate- to large-sized immature cells with large cytoplasm and irregularly shaped nuclei including light chromatin and 2-3 little nucleoli were seen. The immature cells were CD34-negative. Immature cells were accepted as Faggot cells including Auer rods with May-Grünwald Giemsa staining. Bone marrow aspiration revealed 20% blasts and 20% promyelocytes. Thus, the patient’s diagnosis was APL. Spinal lumbosacral MRI showed diffuse bone marrow signal alterations and contrast medium involvement of all vertebrae and iliac bones. A suspicious compression at the L5 level was also reported, but the patient did not have any neurologic deficits. 

Flow cytometric analysis of bone marrow samples identified the presence of an abnormal population of CD13(+)/CD33(+)/CD45(+)/MPO(+)/DR(-)/Tdt(-) cells. Analysis of 25 metaphases showed clonal abnormalities. The karyotype was interpreted as 46,XX,t(8;21)(q22;q22)[1]/46,XX,t(15;17)(q22;q21)[5]/46,XX[24]. Additional fluorescence in situ hybridization (FISH) studies for the PML locus at 15q22 and RARα locus at 17q21 were carried out. Analysis of 100 interphase nuclei showed a hybridization pattern consistent with PML/RARα fusion in 36 (36%) nuclei (nuc ish(PMLx3)(RARAlfax3)(PML conRARAlfax1)[36/100]). In addition, real-time quantitative (RQ) polymerase chain reaction (PCR) analysis with t(15;17) bcr1 transcript revealed 5007 copies (1.78525699%) of fusion transcripts per 1 µg RNA and 305 copies (0.1082556554%) of fusion transcripts per 1 µg RNA with t(15;17) bcr3 transcript. FLT3 ITD and D835 mutations were studied from peripheral blood with the PCR and restriction fragment length polymorphism (PCR-RFLP) method, and both were negative. 

Induction chemotherapy consisting of 3 days of idarubicin (12 mg/m^2^/day) and daily ATRA (45 mg/m^2^/day) was initiated. After the induction therapy, the pancytopenic state improved (Hb: 10^9^ g/L, WBC: 3.6 x 10^9^/L with 2.1 x 10^9^/L absolute count (59.0%) of neutrophils, platelets: 239 x 10^9^/L), and the hemostasis tests returned to normal limits except a mild increase in D-dimer (2.02 mg/mL) and fibrinogen levels (15.6 µmol/L). At the sixth week of treatment, a control bone marrow biopsy was found to be normocellular. In addition, t(15;17) bcr3 and t(8;21) were negative, and t(15;17) bcr1 fusion transcripts were reduced from 5007 (1.78525699%) copies per 1 µg RNA to 40 (0.00062020%). Consolidation treatment including 4 days of idarubicin (5 mg/m^2^/day), ATRA (45 mg/m^2^/day for 15 days), and cytarabine (1 g/m^2^/day for 4 days) was then started. After consolidation therapy, the patient became pancytopenic and had neutropenic fever. She was transferred to the intensive care unit because of acute respiratory distress syndrome-like respiratory problems and multiorgan dysfunction requiring hemodialysis and respiratory support. Despite appropriate antibiotic treatment, multiple red blood cell and platelet transfusions, and inotropic agents, the patient expired 3 monts after the time of APL diagnosis. 

## DISCUSSION

In the APL-93 trial, the incidence of chromosomal abnormalities in addition to t(15;17) was 26%, and trisomy 8 was the most frequent secondary change (46% of the cases with secondary changes) [2]. Additional chromosome rearrangements including t(8;21) and t(15;17) are rarely seen in APL patients treated initially with ATRA plus chemotherapy [11,13,14,15]. Two case studies reported a coexistence of t(8;21) and t(15;17) chromosomal anomalies in their patients at the time of diagnosis, and they concluded that t(8;21) may have been the first event to originate from an early leukemic clone, while t(15;17) was acquired later in the course of the disease [11,13]. Charrin et al. detected 2 clones at the initial phase of an AML including 46,XX,t(15;17) and 46,XX,t(8;21),t(15;17). Relapse occurred after 12 months of complete remission with typical APL syndrome when t(15;17) alone was the most predominant [11]. Movafagh et al. [16] reported a female patient in whom 2 French-American-British (FAB)-specific chromosome aberrations evolved from a single leukemic clone and co-expressed t(15;17) and t(8;21). Recently, in a case of APL-M3v in which complete remission was achieved soon after a course of ATRA plus chemotherapy, a novel t(8;21) chromosomal aberration was detected from 3 to 18 months after initial treatment. Intermittent detection of t(8;21) during periods without ATRA therapy may reflect the antitumor effect of ATRA on M2 leukemic cells and 2 independently growing aberrant stem cell clones in this patient. The authors also suggested that the proportion of M2 leukemic cells at the time of diagnosis should be below the sensitivity level of the nested PCR detection limit, and, after chemotherapy, alteration of bone marrow cell kinetics should trigger t(8;21) via complex mechanisms [12]. 

The prognostic impacts of additional cytogenetic abnormalities in APL patients have been analyzed in a number of studies, and conflicting results were obtained [2,6,7,8,9,10]. Grimwade et al. evaluated 1612 AML patients and found that these cytogenetic changes did not alter the prognosis of patients with favorable karyotypic anomalies such as t(15;17). Trisomy 21 was categorized in the intermediate risk group, while monosomies 5 and 7 had no effects on response rates and overall survival [10]. In the largest study by de Botton et al. [2], patients with only t(15;17) and patients with t(15;17) plus other chromosomal abnormalities were compared. In accordance with the findings of Grimwade et al. [10], additional cytogenetic changes in patients with t(15;17) had no impact on complete remission rate, event-free survival, relapse and overall survival at 2 years. Calabrese et al. suggested that complex chromosome translocations were secondary changes that occurred after standard t(8;21) and t(15;17), thus clarifying the hierarchy of the cytogenetic events [17]. 

Today, ATRA in combination with conventional chemotherapy increases the efficacy of ATRA dramatically and improves the long-term survival of APL patients to 75% [18]. In non-APL AML, the effects of ATRA are less clear. However, there has been a long-standing interest in the clinical use of ATRA for the treatment of AML subtypes other than APL. ATRA has growth inhibitory effects in non-M3 leukemic cells through cytotoxicity and apoptosis [19]. ATRA’s inhibitory roles in non-APL leukemic cells including the HL-60 cell line [20], ovarian carcinoma, neuroblastoma, and germ cell tumors [21] were also reported. Based on these promising scientific data, Schlenk et al. evaluated ATRA in combination with intensive chemotherapy in non-APL elderly AML patients. They suggested that patients with the genotype mutant NPM1 without FLT3 ITD who had been randomized to the ATRA arm had a significantly better relapse-free and overall survival compared to patients with the same genotype who had not been randomized to the ATRA arm. They considered that ATRA may exert its effect by down-regulation or by posttranslational modification of the antiapoptotic protein bcl-2 [22]. Four cases of AML that were initially misdiagnosed as APL based on FAB classification were successfully treated with ATRA alone. Three of these patients achieved complete response (CR), but all of them relapsed early. Their diagnoses were changed to t(8;21) AML based on karyotype analysis. One of the 3 patients who achieved CR had a decrement of the AML1-ETO fusion gene from 92% to 0% after 10 days of ATRA treatment. However, prior use of arsenic trioxide in the same patient did not alter the AML1-ETO fusion gene in the bone marrow cells according to FISH analysis. Interestingly, one patient who had a temporary response (without CR) with ATRA achieved CR after a combined treatment including daunorubicin and cytarabine. The presence of both AML1-ETO and PML-RARα fusion genes were detected by RT-PCR in this patient [23]. The authors suggested that AML1-ETO and PML-RARα recruit a multiprotein complex containing histone deacetylases (HDACs) on crucial myeloid differentiation via several co-repressors, which leads to a cell differentiation block [24]. In addition, oligomerization and formation of high-molecular-weight complexes by AML1-ETO and PML-RARα play a critical role in the aberrant recruitment of HDAC activity [25]. 

Our case showed the typical APL morphology and co-expression of t(8;21) and t(15;17), which was detected by conventional cytogenetic analysis. RQ-PCR technology has recently reached a level of sensitivity, accuracy, and practical ease that supports its use as a routine bioinstrumentation for gene level measurement [26]. However, in our patient, t(8;21) was determined by cytogenetic analysis, but not by RQ-PCR. FLT3 ITD and D835 mutations were both negative. Classic AIDA induction chemotherapy [27,28] was administered to the patient. She went into remission after induction chemotherapy. t(15;17) bcr3 and t(8;21) became negative, and t(15;17) bcr1 fusion transcripts were reduced from 5007 (1.78525699%) copies per 1 µg RNA to 40 (0.00062020%) according to RQ-PCR. Consolidation treatment including 4 days of idarubicin (5 mg/m2/day), ATRA (45 mg/m2/day for 15 days), and cytarabine (1 g/m2/day for 4 days) was then started. 

Co-expression of t(8;21) and t(15;17) is rarely seen in APL patients. The role of chromosome translocations other than t(15;17) in APL is still unclear. The current literature data support the concept that patients harboring t(15;17) with any other additional chromosomal abnormality a similarly favorable prognosis as patients with t(15;17) alone. Combined use of conventional cytogenetics, FISH [29], and PCR should increase the detection of additional chromosomal abnormalities either in diagnosis or in the treatment period of APL. Combination therapy with ATRA, Ara-C, and anthracycline should be an appropriate choice to improve the prognosis in these patients. Further studies are required to clarify the clinical features and prognosis with complex translocations. 

## CONFLICT OF INTEREST STATEMENT

The authors of this paper have no conflicts of interest, including specific financial interests, relationships, and/or affiliations relevant to the subject matter or materials included.
